# Acute Inflammatory Demyelinating Polyradiculoneuropathy Presenting With Multiple Cranial Nerve Palsies and Total Ophthalmoplegia: The Importance of Accurate Diagnosis and Timely Immunotherapy

**DOI:** 10.1155/carm/3658834

**Published:** 2026-08-13

**Authors:** Niswah Silmi Fatimah, Abdulloh Machin

**Affiliations:** ^1^ Department of Neurology, Faculty of Medicine, Airlangga University, Surabaya, Indonesia, unair.ac.id; ^2^ Department of Neurology, Airlangga University Hospital, Surabaya, Indonesia; ^3^ Department of Neurology, Dr. Soetomo Regional General Hospital, Surabaya, Indonesia

**Keywords:** AIDP, case report, Guillain–Barré syndrome, IVIG, total ophthalmoplegia

## Abstract

Guillain–Barré syndrome (GBS) is an immune‐mediated disorder of the peripheral nervous system characterized by progressive weakness and often triggered by infections. GBS has an estimated mortality rate of approximately 5%, with a worldwide incidence of 0.81–1.91 cases per 1,00,000 person‐years. This case reported a 23‐year‐old man presented with progressive weakness in all four extremities. An initial neurological examination revealed bilateral total ophthalmoplegia, ptosis on both eyes, bilateral facial palsy lower motor neuron (LMN) type, dysarthria, dysphagia, flaccid tetra‐paresis, gloves, and stocking paresthesia. Lumbar puncture showed cytoalbumin dissociation. Nerve conduction studies showed a demyelinating sensorimotor polyradiculoneuropathy lesion. The patient received intravenous immunoglobulin (IVIG) treatment 0.4 g/kg/day for 5 days, physical rehabilitation therapy, nutritional support, multidisciplinary care involving anesthesiology and intensive care teams, and other symptomatic treatments. At five‐month follow‐up, the patient demonstrated complete recovery. GBS typically presents as an acute ascending sensorimotor neuropathy; however, atypical presentations may occur. Extensive cranial nerve involvement—including complete ophthalmoplegia, bilateral facial palsy, and bulbar dysfunction—in AIDP is uncommon and may mimic other GBS spectrum disorders. Early recognition, prompt immunotherapy, and careful electrophysiological evaluation are essential for optimizing patient outcomes. Because electrodiagnostic classification of GBS may evolve over time, serial nerve conduction studies should be considered whenever feasible to improve subtype classification and diagnostic confidence.

## 1. Introduction

Guillain–Barré syndrome (GBS) is an immune‐mediated disease of the peripheral nervous system that is triggered by infections [1, 2]. GBS has an estimated mortality rate of 5%, with a worldwide incidence of 0.81–1.91 cases per 100,000 person‐years [3, 4]. Classic GBS typically presents as an acute‐onset ascending sensorimotor neuropathy and flaccid paralysis [1, 4]. There are several variants of GBS such as acute inflammatory demyelinating polyradiculoneuropathy (AIDP), acute motor axonal neuropathy (AMAN), acute motor sensory axonal neuropathy (AMSAN) [1], Miller Fisher syndrome (MFS), and Bickerstaff brainstem encephalitis (BBE) [1, 5].

## 2. Case Presentation

### 2.1. Patient Information

A 23‐year‐old man presented with progressive weakness in all extremities. The symptoms began five days prior, when the patient complained of fever and diarrhea. The patient initially sought treatment at a local primary health facility, given medication to take home, and the symptoms improved. The next day, the patient felt weakness in both lower extremities. The patient was then taken to the referral hospital. Three days later, the symptoms worsened, with weakness in both lower extremities, followed by upper extremities weakness, slurred speech, and difficulty opening both eyelids. The patient also complained of being unable to eat or drink and tingling in the tips of the fingers and toes.

### 2.2. Clinical Findings

**FIGURE 1:**
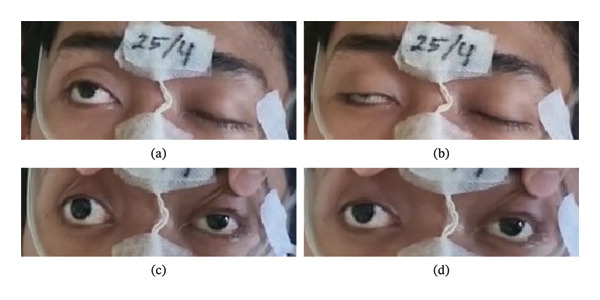
Clinical examination demonstrating ocular motor abnormalities—(a) ptosis ODS (OS > OD) at primary gaze; (b) incomplete left eyelid closure (lagophthalmos) OD; (c) attempted rightward gaze demonstrating complete horizontal ophthalmoplegia; (d) attempted leftward gaze demonstrating persistent bilateral limitation of extraocular movements. These findings are consistent with severe external ophthalmoplegia. Abbreviations: OD = oculus dexter (right eye); OS = oculus sinister (left eye). Clinical findings (as mentioned in section Case Presentation, Clinical Findings, Paragraph 1, Line 3).

The neurological examination revealed bilateral total ophthalmoplegia, ptosis oculi dextra et sinistra (ODS) with OS > OD (Figure 1), bilateral facial palsy LMN type, lingual and labial dysarthria, and dysphagia (Table 1). Motor strength of the upper extremity was symmetrical Medical Research Council (MRC) sum Score 2 in the arm abduction and elbow flexion, and MRC Grade 3 in the wrist extension. Motor strength of the lower extremity was symmetrical with MRC Grade 2 in the hip flexion and knee extension, and MRC Grade 3 in ankle dorsiflexion. Biceps physiological reflex (BPR) and triceps physiological reflex (TPR) were decreased + 1, and knee physiological reflex (KPR) and ankle physiological reflex (APR) were decreased with a value of zero. The sensory examination revealed gloves and stocking paresthesia. The Hughes functional grading scale was four, with an Erasmus GBS Respiratory Insufficiency Score (EGRIS) of six.

**TABLE 1:** Multiple cranial nerve involvement of these findings suggest extensive cranial nerve root involvement.

Nerve	Symptoms
III	Ptosis
III, IV, VI	Total ophthalmoplegia
VII	Bilateral facial palsy
IX‐X	Dysphagia
XII	No clinical involvement

*Note:* Laboratory result (as mentioned in section Case Presentation, Diagnostic Assessment, paragraph 1, Line 2).

### 2.3. Diagnostic Assessment

Lumbar puncture performed on Day 6 of symptom onset showed cytoalbumin dissociation (Table 2). Nerve conduction studies on Day 7 showed a demyelinating sensorimotor polyradiculoneuropathy lesion (Tables 3, 4, 5, and 6).

**TABLE 2:** The CSF analysis demonstrating albumin cytologic dissociation, characterized by a markedly elevated protein concentration of 152.33 mg/dL (reference range: 15–40 mg/dL) with normal white blood cell count of 2 cells/µL (< 5 cells/µL), while glucose levels remained within normal limits.

Parameter	Results	Reference
Appearance (CSF)	Colorless, clear, no clots	Colorless, clear, no clots
Others (CSF)	pH 8	Negative
WBC‐BF (CSF)	2/µL	< 5
RBC‐BF (CSF)	0/µL	—
MN# (CSF)	1/µL	4.3–10
PMN# (CSF)	1/µL	—
MN% (CSF)	50%	—
PMN% (CSF)	50%	—
Cell count (CSF)	2/µL	< 2000
Glucose (CSF)	63.6 mg/dL	< 100
Total protein	152.33 mg/dL	15–40

*Note:* These findings are typical of Guillain–Barré syndrome. EMG‐NCV result (as mentioned in section Case Presentation, Diagnostic Assessment, Paragraph 1, Line 3).

**TABLE 3:** Bilateral sural sensory nerve conduction study demonstrating the absence of the left sural sensory nerve action potential (NR).

Stim site	NR	Peak (ms)	Norm peak (ms)	P‐T amp (µV)	Norm P‐T amp	Site1	Site2	Delta‐P (ms)	Dist (cm)	Vel (m/s)	Norm vel (m/s)
*Left sural antisensory (lat mall)*											
Calf	NR		< 4,0		> 10	Calf	Lat mall		14,0		

*Right sural antisensory (lat mall)*											
Calf		2,4	< 4,0	11,5	> 10	Calf	Lat mall	2,4	14,0	58	

*Note:* This sensory abnormality should be interpreted together with the motor nerve conduction findings (Tables 4, 5, and 6). Peak, peak latency; P‐T Amp, peak‐to‐peak amplitude; Norm peak, normal reference peak latency; Norm P‐T Amp, normal reference peak‐to‐peak amplitude; Stim site, stimulation site; Site1 and Site2, recording and stimulation sites; Delta‐P, latency difference; Dist, distance; Vel, conduction velocity; m/s, meters per second; ms, milliseconds; µV, microvolts; Lat Mall, lateral malleolus.

Abbreviation: NCS, nerve conduction study; NR, no response.

**TABLE 4:** Motor nerve conduction study (NCS) revealed bilateral prolongation of distal motor latency in the fibular nerves, with preserved motor conduction velocities and mildly reduced compound muscle action potential amplitudes in the left fibular nerve tibial motor responses were relatively preserved.

Stim site	NR	Onset (ms)	Norm onset (ms)	O‐P amp (mV)	Norm O‐P amp	Site1	Site2	Delta‐0 (ms)	Dist (cm)	Vel (m/s)	Norm vel (m/s)
*Left fibular motor (ext dig brev)*											
Ankle	5,2		< 4	3,1	> 2	B Fib	Ankle	6,1	33,0	54	> 38
B Fib	11,3			2,8		Poplt	B fib	1,8	8,0	44	> 40
Poplt	13,1			1,2							

*Right fibular motor (ext dig brev)*											
Ankle	4,3		< 4	3,4	> 2	B Fib	Ankle	7,1	33,0	46	> 38
B fib	11,4			3,6		Poplt	B fib	1,4	8,0	57	> 40
Poplt	12,8			3,0							

*Left tibial motor (abd hall brev)*											
Ankle	3,8		< 4	10,0	> 7	Knee	Ankle	8	39,0	49	> 35
Knee	11,8			8,2							

*Right tibial motor (abd hall brev)*											
Ankle	3,8		< 4	11,5	> 7	Knee	Ankle	8,9	39,0	44	> 35
Knee	12,7			11,1							

*Note:* Together with the sensory nerve conduction abnormalities (Table 3) and absent H‐reflexes (Table 6).

**TABLE 5:** F‐wave studies of the bilateral tibial nerves demonstrated normal F‐wave latencies on both sides, indicating preserved proximal motor conduction.

Examined nerve	F‐lat (ms)	Lat norm (ms)	L‐R F‐lat (ms)	L‐R lat norm
*Left tibial (abd hallucis)*				
	48,75	< 50	0,78	< 5,7

*Right Tibial (Abd Hallucis)*				
	49,53	< 50	0,78	< 5,7

*Note:* These findings should be interpreted together with the motor and sensory nerve conduction studies. Lat Norm, normal reference latency; Abd Hallucis, abductor hallucis; F‐Lat, F‐wave latency; L‐R F‐Lat, ms, milliseconds; left‐to‐right F‐wave latency difference; L‐R Lat Norm, normal reference value for left‐to‐right F‐wave latency difference; NR, nerve examined.

**TABLE 6:** H‐reflex studies demonstrating bilateral absent H‐reflexes, supporting proximal demyelination.

Examined nerve	H‐lat (ms)	L‐R H‐lat (ms)	L‐R lat norm
*Left tibial (Gastroc)*			
NR			< 2,0

*Right tibial (Gastroc)*			
NR			< 2,0

*Note:* L‐R H‐Lat, left‐to‐right H‐reflex latency difference; H‐Lat, H‐reflex latency; ms, milliseconds; L‐R Lat Norm, normal reference value for the left‐to‐right latency difference; Gastroc, gastrocnemius muscle.

Abbreviation: NR, no response.

### 2.4. Therapeutic Intervention

The patient received IVIG treatment at a dose of 0.4 g/kg body weight for 5 days (total 2 gr/kg), starting on the 7th day. The patient also received physical rehabilitation therapy, nutritional support, and multidisciplinary care including anesthesiologists and intensive care (standby), as well as other symptomatic treatments.

### 2.5. Follow‐Up and Outcomes

The patient was then discharged with improved clinical condition on the onset of Day 15, where upper and lower extremities motor function was rated MRC Grade 4. However, no significant improvement was seen in the involvement of other cranial nerves. The patient’s modified EGOS score upon discharge was four. Follow‐up was conducted 2 weeks after the patient was discharged, with upper and lower extremities motor strength rated at MRC Grade 5, and the patient began to walk. Three months after admission, the patient was able to run lightly, although slurred speech and slight eyelid closure were still present. Four months after admission, the patient was able to ride a motorcycle, with residual slurred speech and facial movements that had not fully improved. Five months after admission, the patient was able to perform usual daily activities. Three years after admission, no recurrent episodes of weakness were observed in this patient.

## 3. Discussion

The incidence of GBS in Asia is 0.44–3.25/100.000 person/years. GBS is more common in men than in women, with a ratio of 1.5:1 [5]. GBS can progress rapidly, with peak disability typically reached within two weeks. Autonomic dysfunction—such as cardiac arrhythmias and blood pressure instability—occurs in 3–10% of cases and contributes to mortality [6]. The underlying pathophysiology involves both T‐cell– and B‐cell–mediated immune responses, activation of the complement system, the presence of antiganglioside antibodies, and molecular mimicry. In this process, a preceding infection exposes antigens that share structural similarities with peripheral nerve components, leading to an unintended immune attack on nerve tissue. Additionally, increased production of matrix metalloproteinases (MMP‐9 and MMP‐2) has been observed in GBS and is thought to contribute to nerve damage through lysis of the basement membrane [6].

**FIGURE 2:**
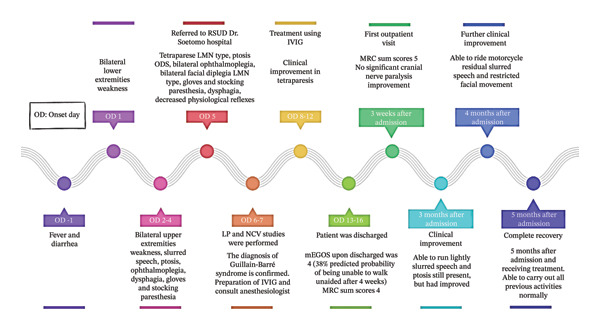
Timeline of the patient as described in Case Presentation. Abbreviations: OD, onset‐day; LMN, lower motor neuron; LP, lumbar puncture; NCV, nerve conduction velocity; IVIG, intravenous immunoglobulin; MRC, Medical Research Council; mEGOS, modified Erasmus GBS outcome score. Patient timeline (as mentioned in section Discussion, Paragraph 2, Line 9).

Clinicians began to suspect GBS in this patient due to rapidly progressive bilateral limb weakness, followed by cranial nerve involvement within 4 days of onset. The presence of cytoalbumin dissociation on the CSF examination and demyelinating features on nerve conduction studies supported the diagnosis of GBS. The patient fulfilled the electrodiagnostic criteria for AIDP according to the Hadden classification [7]. The diagnosis of AIDP was favored over MFS due to the absence of ataxia and over BBE due to preserved consciousness [8]. However, an axonal GBS subtype (AMAN) with reversible conduction failure cannot be completely excluded in the absence of serial electrophysiological studies and antiganglioside antibody testing [7, 9]. A timeline of clinical events is presented in Figure 2. There was no autonomic dysfunction observed during hospitalization or any treatment‐related fluctuation (TRF) after given treatment. The patient expressed satisfaction with the multidisciplinary care received and was able to return to his usual daily activities within five months.

Cranial nerve involvement occurs in 50–75% of patients with GBS. Among these, bulbar palsy is the most frequently observed manifestation (49.2%), presenting with dysphagia and dysphonia, followed by facial palsy (46%), and the rarest involvement including tongue paralysis (10%) and ophthalmoplegia (6.5%) [8, 10, 11]. The extensive cranial nerve involvement observed in this patient may reflect demyelination affecting the cranial nerve roots at the brainstem exit zones, which are particularly vulnerable to immune‐mediated injury in AIDP.

Nerve conduction studies in GBS will show demyelination with or without axonal loss, prolonged distal motor latency, prolonged F wave latency, temporal dispersion of CMAP, the presence of conduction block, and the absence of H‐reflexes [6, 12].

This case highlights that although cranial nerve involvement is relatively common in GBS, extensive involvement including complete ophthalmoplegia, bilateral facial palsy, and bulbar dysfunction in the AIDP subtype remains uncommon and may mimic other GBS variants such as MFS or BBE. Although transient distal motor latency prolongation has been described in early AMAN due to reversible conduction failure, the electrophysiological findings in our patient fulfilled the Hadden electrodiagnostic criteria for AIDP based on prolonged distal motor latencies and supportive electrophysiological abnormalities. Nevertheless, because upper‐limb nerve conduction studies, serial electrophysiological examinations, and antiganglioside antibody testing were not performed, an axonal GBS subtype with reversible conduction failure cannot be completely excluded.

### 3.1. Limitation

This case report has several limitations. First, upper‐limb nerve conduction studies, serial electrophysiological examinations, and antiganglioside antibody testing were not performed, limiting definitive differentiation between AIDP and axonal GBS subtypes, particularly AMAN with reversible conduction failure. Second, as this is a single case report, the findings cannot be generalized to all patients with GBS. Nevertheless, the detailed clinical presentation, cerebrospinal fluid findings, and electrophysiological evaluation provide valuable educational insights into an uncommon presentation of GBS with extensive cranial nerve involvement.

## 4. Conclusion

GBS typically presents as an acute ascending sensorimotor neuropathy; however, atypical presentations may occur. Extensive cranial nerve involvement—including complete ophthalmoplegia, bilateral facial palsy, and bulbar dysfunction—in AIDP is uncommon and may mimic other GBS spectrum disorders. Early recognition, prompt immunotherapy, and careful electrophysiological evaluation are essential for optimizing patient outcomes. Because electrodiagnostic classification of GBS may evolve over time, serial nerve conduction studies should be considered whenever feasible to improve subtype classification and diagnostic confidence.

## Author Contributions

Niswah Silmi Fatimah conceived the report, collected and analyzed the clinical data, performed the literature review, prepared the figures and tables, drafted the manuscript, and revised the manuscript. Abdulloh Machin supervised the study, contributed to the interpretation of the clinical findings, critically reviewed and revised the manuscript for important intellectual content, and provided overall academic guidance.

## Funding

No funding was received for the preparation of this case report.

## Disclosure

Both authors approved the final manuscript and agreed to be accountable for all aspects of the work.

## Ethics Statement

This case report was conducted in accordance with the ethical principles of the Declaration of Helsinki. Written informed consent was obtained from the patient for publication of this report and accompanying images. According to the policy of our institution, ethical approval was not required for the publication of a single anonymized case report.

## Conflicts of Interest

The authors declare no conflicts of interest.

## Supporting Information

Additional supporting information can be found online in the Supporting Information section.

## Supporting information


**Supporting Information** This case report was prepared in accordance with the CARE reporting guidelines.

## Data Availability

Data sharing is not applicable to this article as no datasets were generated or analyzed during the current study.
