# Supplementary material for: Acute Inflammatory Demyelinating Polyradiculoneuropathy Presenting With Multiple Cranial Nerve Palsies and Total Ophthalmoplegia: The Importance of Accurate Diagnosis and Timely Immunotherapy
Source: Case Rep Med. 2026 Aug 13;2026:3658834. doi: 10.1155/carm/3658834 (PMC13470610; doi:10.1155/carm/3658834)
Supplement: Supplementary file 1 [file CARM-2026-3658834-s001.pdf]

CARE case report guidelines

CARE Checklist of information to include when writing a case report

(cc) BY-NC-ND

Topic	Item	Checklist item description	Reported on Line
Title	1	The diagnosis or intervention of primary focus followed by the words "case report"	in Title
Key Words	2	2 to 5 key words that identify diagnoses or interventions in this case report, including "case report"	in Keywords
Abstract (no references)	3a	Introduction: What is unique about this case and what does it add to the scientific literature?	Abstract, line 11
	3b	Main symptoms and/or important clinical findings	Abstract, Line 4-8
	3c	The main diagnoses, therapeutic interventions, and outcomes	Abstract, line 8 - 10
	3d	Conclusion—What is the main "take-away" lesson(s) from this case?	Abstract line 11-14
Introduction	4	One or two paragraphs summarizing why this case is unique (**may include references**)	Introduction
Patient Information	5a	De-identified patient specific information.	Case presentation, part patient information
	5b	Primary concerns and symptoms of the patient.	Case presentation, part patient information
	5c	Medical, family, and psycho-social history including relevant genetic information	Case presentation, part patient information
	5d	Relevant past interventions with outcomes	Case presentation, part patient information
Clinical Findings	6	Describe significant physical examination (PE) and important clinical findings.	Case presentation, part clinical findings
Timeline	7	Historical and current information from this episode of care organized as a timeline	Figure 2
Diagnostic Assessment	8a	Diagnostic testing (such as PE, laboratory testing, imaging, surveys).	Case presentation, part diagnostic assessment
	8b	Diagnostic challenges (such as access to testing, financial, or cultural)	Discussion, part limitation
	8c	Diagnosis (including other diagnoses considered)	Discussion, paragraph 2
	8d	Prognosis (such as staging in oncology) where applicable	Case presentation, part follow-up and Outcomes
Therapeutic Intervention	9a	Types of therapeutic intervention (such as pharmacologic, surgical, preventive, self-care)	Case presentation, part therapeutic intervention
	9b	Administration of therapeutic intervention (such as dosage, strength, duration)	Case presentation, part therapeutic intervention
	9c	Changes in therapeutic intervention (with rationale)	Case presentation, part therapeutic intervention
Follow-up and Outcomes	10a	Clinician and patient-assessed outcomes (if available)	Case presentation, part follow-up and outcomes
	10b	Important follow-up diagnostic and other test results	Table 2 - table 6
	10c	Intervention adherence and tolerability (How was this assessed?)	Case presentation, part therapeutic intervention
	10d	Adverse and unanticipated events	Case presentation, part therapeutic intervention
Discussion	11a	A scientific discussion of the strengths AND limitations associated with this case report	Discussion, part limitation
	11b	Discussion of the relevant medical literature **with references**.	Discussion
	11c	The scientific rationale for any conclusions (including assessment of possible causes)	Discussion
	11d	The primary "take-away" lessons of this case report (without references) in a one paragraph conclusion	Conclusion
Patient Perspective	12	The patient should share their perspective in one to two paragraphs on the treatment(s) they received	Discussion, paragraph 2, last line
Informed Consent	13	Did the patient give informed consent? Please provide if requested	Yes ☑ No ☐
